# Income disparities in smoking cessation and the diffusion of smoke-free homes among U.S. smokers: Results from two longitudinal surveys

**DOI:** 10.1371/journal.pone.0201467

**Published:** 2018-07-27

**Authors:** Maya Vijayaraghavan, Tarik Benmarnhia, John P. Pierce, Martha M. White, Jennie Kempster, Yuyan Shi, Dennis R. Trinidad, Karen Messer

**Affiliations:** 1 Division of General Internal Medicine, Zuckerberg San Francisco General Hospital, University of California San Francisco, San Francisco, CA, United States of America; 2 Division of Population Sciences, Moores Cancer Center, University of California San Diego, La Jolla, CA, United States of America; 3 Department of Family Medicine and Public Health, University of California San Diego, La Jolla, CA, United States of America; 4 Climate, Atmospheric Science & Physical Oceanography, Scripps Institution of Oceanography, La Jolla, CA, United States of America; Harvard University, UNITED STATES

## Abstract

**Background:**

Lower rates of successful quitting among low-income populations in the United States may be from slower dissemination of smoke-free homes, a predictor of cessation.

**Objectives:**

To explore the role of smoke-free homes in cessation behavior across income levels.

**Participants:**

Current smokers who were ≥18 years and who participated in the longitudinal 2002–2003 (n = 2801) or 2010–2011 (n = 2723) Tobacco Use Supplements to the Current Population Survey.

**Measurements:**

We categorized income as multiples of the federal poverty level (FPL) (<300% FPL versus ≥300% FPL). We examined the association of smoke-free homes with 1+day quit attempts and 30+days abstinence at 1-year follow-up. We then conducted a mediation analysis to examine the extent that smoke-free homes contributed to income disparities in 30+days abstinence.

**Results:**

Between the two surveys, heavy smoking (≥ 1 pack/day) declined by 17%, and smoking prevalence declined by 15% among those with higher-incomes (>300%FPL). Although similar in 2002, the prevalence of smoke-free homes was 33% lower among individuals living <300% FPL than those living ≥300% FPL. Although the quit attempt rate was similar, the 30+days abstinence rate was higher in the 2010–11 cohort than in 2002–3 cohort (20.6% versus 15.5%, p<0.008). Whereas smoking ≥ 1 pack/ day was associated with lower odds of 30+days abstinence (Adjusted odds ratio [AOR] 0.7; 95% CI 0.5–0.9), having a higher income (AOR 1.9, 95% CI 1.4–2.6) and a smoke-free home (AOR 1.6, 95% CI 1.2–2.1) were associated with greater odds of 30+day abstinence. Differential changes in smoke-free homes across income groups between the two surveys contributed to 36% (95% CI 35.7–36.3) of the observed income disparity in 30+days abstinence.

**Conclusions:**

Increasing the diffusion of smoke-free homes among low-income populations may attenuate at least a third of the income disparities in smoking cessation, highlighting the need for interventions to increase adoption of smoke-free homes among low-income households.

## Introduction

While the decline in cigarette smoking in the United States (U.S.) population has been a considerable public health success story,[1] the gradient by income has been marked.[2] The effect of a much higher smoking prevalence in low income groups is seen in a disproportionate burden of tobacco-related chronic diseases.[1, 3, 4] One reason for these higher smoking rates has been lower rates of successful cessation among lower income smokers compared to higher income smokers.[5, 6]

A smoke-free home, where smoking is not allowed anywhere inside the home, is one of the major predictors of successful cessation. Evidence from cross-sectional and longitudinal population-based surveys with nationally representative samples has shown that smoke-free homes reduce consumption,[7, 8] increase successful cessation,[7, 8] and reduce relapse to smoking.[7] Although smoke-free homes are more prevalent among lighter smokers, the benefit of smoke-free homes is seen among all smokers, including moderate-to-heavy smokers.[7] Smoke-free homes are more prevalent in states with strong clean indoor air laws, supporting the normative effects of these laws.[9] Smoke-free homes are more common in households without other smokers and those with children.[10] They are much less common in low-income households,[11] reflecting differential exposure to clean indoor air laws in low-income communities.[12]

The discussion on smoke-free homes is particularly salient given that the U.S. Department of Housing and Urban Development (HUD) enacted a rule, effective July 2018, that mandated all public housing authorities (PHAs) to implement indoor no-smoking policies. [13] Such policies, in combination with educational campaigns directed toward property management companies,[14] have the potential to substantially increase exposure to smoke-free home rules among the very populations that are disproportionately affected by tobacco use. In doing so, smoke-free homes could reduce the income disparity in cessation.[15]

Aside from differences in exposure to smoke-free homes, across the US, there are also large differences in the level of tobacco control action implemented by state governments. Common tobacco control policies include: a) increasing state cigarette taxes (in April 2017, state taxes varied from $0.37/pack to $4.35/pack),[16] b) passing smoke-free workplace legislation to restrict non-smoker exposure to secondhand smoke,[17] and c) allocating expenditures for tobacco control programs as recommended by the Centers for Disease Control and Prevention’s (CDC) *Best Practices for Comprehensive Tobacco Control Programs*.[18] Such policies have the potential to impact both the proportion of smokers who make a quit attempt as well as the proportion who successful quit smoking.[8, 11, 19–22] Additionally, guideline-recommended treatments for tobacco dependence (e.g. pharmacotherapy and behavioral counseling) have been shown to be effective in increasing cessation in clinical trials.[23]

In this study, we explored factors that could explain the lower cessation rates among low-income smokers, focusing on the impact of smoke-free homes. We used the 2002/3 and 2010/11 longitudinal subsamples of the Tobacco Use Supplement to the Current Population Survey (TUS-CPS) to examine changes in the smoking population over time and the diffusion of smoke-free homes across income levels and over time. During this time we expected that the prevalence of smoke-free homes would increase, while the proportion of states with tobacco control programs funded at the levels recommended by the CDC Best Practices would decrease.[18] We examined the independent association of smoke-free homes with 1+-day quit attempts and 30+-day abstinence at 1-year follow-up, adjusting for smoking behavior, tobacco control policy (i.e. state cigarette price and CDC state tobacco control program expenditures), and receipt of cessation services. We hypothesized that the slower diffusion of smoke-free homes among low-income smokers may, in part, explain the lower cessation rates in this population, and conducted mediation analyses to examine these associations.

## Methods

### Data source

The Current Population Survey (CPS), conducted by the Census Bureau, uses a complex multistage probability sample to collect information from a nationally representative, non-institutionalized population.[24] Once enrolled in the sample, members of households are interviewed for 4 consecutive months, rested for 8 months, and re-interviewed for another 4 months before exiting the sample.[24] Tobacco Use Supplements (TUS), coordinated by the National Cancer Institute,[25] have been added to the CPS at regular intervals. When supplements are conducted on the same month in consecutive years, there will be an overlap sub-sample that constitutes a longitudinal study. This was done in February 2002 and 2003 and again in May 2010 and 2011. The TUS-CPS allows both proxy and self-response and the response rate was 83.7% in February 2002 and 82.2% in May 2010, respectively. The follow-back response rate was 66.1% in 2003 and 66.8% in 2011. All data are weighted to represent the national population. In this analysis, we include adult (18+ years) respondents who self-reported smoking behavior on both surveys and who were current smokers at the baseline survey. Less than 7% of the sample had missing income data (n = 183) at baseline; we included these individuals in the analyses. This resulted in 2801 participants in 2002/3 and 2723 in 2010/11.

### Measures

#### Cigarette smoking measures at baseline

Ever smokers are respondents who have smoked at least 100 cigarettes in their lifetime. Current smokers are those ever-smokers who reported smoking every day or somedays. Current smokers reported their usual cigarette consumption on days that they smoked, which we used to estimate average daily cigarette consumption. We categorized consumption as 0 to 9 cigarettes per day, 10–19 cigarettes per day, and ≥ 20 cigarettes per day.

#### Cigarette smoking cessation measures at follow-up

On the follow-up survey, participants were asked if they had made an intentional quit attempt lasting at least one day in the past year. Non-smokers at follow-up were asked how long it was since they had completely quit smoking cigarettes. As it is has been established that at least half of those with a quit attempt will relapse in the first month, we used 30+-days abstinence as an early marker of successful cessation.[26]

#### Smoke-free homes

Participants were asked their rules on smoking within the home and only those who indicated that “no one is allowed to smoke anywhere inside your home” were categorized as having a smoke-free home at baseline.

#### Policy-level interventions

State tax data [27] were adjusted to 2011 dollars using the annual average consumer price index [28] and we categorized states in tertiles separately for 2002 and 2010. We obtained updated estimates from the State Tobacco Control Expenditure Database,[29] which includes spending on tobacco surveillance and evaluation efforts, anti-tobacco advertising campaigns, and tobacco control programs administration and management costs. State expenditures in 2002 and 2010 were converted into a percentage of the CDC-recommended level [18] and categorized into quartiles.

#### Cessation strategies

Participants were asked whether they had received a doctor’s advice to quit smoking in the past 12 months, and whether they had used a pharmaceutical aid (i.e. any nicotine replacement therapy, bupropion, or varenicline) for their most recent quit attempt. We dichotomized responses as none versus any use of a pharmaceutical aid during the last quit attempt.

#### Demographic and other variables

We included the following demographic measures: age group (18–24 years, 25–44 years, 45–64 years, and ≥ 65 years), sex, race/ethnicity (Non-Hispanic White, African American, Asian/PI, Hispanic/Latino, and Other), and education (less than high school, high school graduate, some college, and college graduate). We used self-reported income and household size to classify respondents into the following income groups relative to the federal poverty level (FPL): < 100% FPL, 100%-199% FPL, 200%-299% FPL, ≥ 300% FPL.[30] For subsequent analyses, we dichotomized poverty status to low-income (<300% FPL) and higher income (≥ 300% FPL) based on the demographic distribution of the sample.

#### Statistical analysis

Variance estimates were calculated using replicate weights constructed using Fay’s balanced repeated replications and supplied by the Census Bureau.[24, 31] Analyses were conducted in SAS 9.4 (SAS Institute, Cary, NC). Using weighted proportions (PROC SURVEYFREQ), we explored differences in sample characteristics between survey years and by income. We used multivariable logistic regression to examine the adjusted association of smoke-free homes with 1+days quit attempts and 30+days abstinence. We then examined potential interactions between: i) smoke-free homes and consumption and ii) smoke-free homes and income. All models were adjusted for a priori identified confounders including demographics, smoking behavior, tobacco control policy (i.e. cigarette taxes, and state tobacco control expenditures), and receipt of cessation services (i.e. doctor’s advice to quit, and use of pharmaceutical aids). We pooled the data from the 2 longitudinal samples to increase power, as we have done in prior studies. [32, 33]

To identify to what extent an increase in smoke-free homes between the two surveys contributed to income disparities in 30+days abstinence, we conducted causal mediation analysis (2 way decomposition), where we decomposed the total effects of the predictor on the outcome into controlled direct and natural indirect effects.[34] This approach is based on the counterfactual framework. [34] The Total Effect (TE) represents the association between income level and 30+days abstinence (which quantifies the income disparity). The Controlled Direct Effect (CDE) represents the effect of income on 30+days abstinence, after hypothetically intervening to fix the level of smoke-free homes to a specific value (here: no change). The Natural Indirect Effect (NIE) represents the change in 30+days abstinence when income is held constant, and smoke-free homes changes to what it would have taken for a change from low to high income. In this approach, we considered as assumptions for the interpretation for TE, NIE and CDE, that there were no unmeasured confounders and that there was no mediator-outcome confounder that was affected by the exposure. Also, we did not include any interaction term in these analyses, so that the natural indirect effects and controlled indirect effects can be interpreted similarly. We estimated NIE and CDE by fitting two consecutive logistic regressions respectively: the outcome model and the mediator model. All models were adjusted for the confounders described above. The total effect is the product of CDE and NIE (OR^CDE^ x OR^NIE^). TEs, CDEs, and NIEs and their 95% confidence intervals were computed using bootstrapping procedures with 240 replications. Finally, we also calculated the proportion mediated in % as follows: OR^CDE^(OR^NIE^-1) / (OR^CDE^OR^NIE^– 1).

## Results

### Differences in baseline characteristics between the two longitudinal surveys

Although the two nationally representative surveys were 9 years apart, there were no significant differences in the proportion of smokers by gender, race-ethnicity or by education **(Table 1).** However, in 2010, smokers were significantly more likely to be older than they were in 2002 (p<0.0001) with declines of 7.4% among 18–29 year olds and 14.2% among 30–49 year olds and a very large increase (40.2%) in the proportion who were aged 50–64 years. Smokers in 2010 were more likely to have lower incomes than those in 2002 (p<0.0008). The proportion of smokers with incomes below the FPL increased to 20% of all smokers in 2010 and there were a further 25% of smokers with incomes less than 200% of FPL. These increases were offset by a 15% decline in smokers with incomes ≥ 300% of the FPL. There was also a statistically significant change in consumption, with a 13% increase in smokers who smoked 0 to 9 cigarettes per day and a 17% decline in smokers who smoked ≥ 20 cigarettes per day (p<0.0005). There was a 62% increase in households with a home smoking ban (p<0.0001), a 63% increase in smokers living in states in the middle tertile for cigarettes taxes (p<0.0001) and a corresponding 42% decrease in smokers living in states with the highest taxes. There was a 49% increase in smokers living in states with the lowest tobacco control expenditures (p<0.0001).

**Table 1:** Sample characteristics among current smokers by baseline survey year (TUS-CPS).

	2002/2003 N = 2801		2010/2011 N = 2723		% Change	P-Value ^c^
	N	% (SE)	N	% (SE)		
**Age**						**< .0001**
18–29 years	379	24.0 (1.1)	346	22.2 (1.2)	-7.4	
30–49 years	1423	47.3 (1.1)	1159	40.6 (1.2)	-14.2	
50–64 years	722	20.7 (0.9)	954	29.0 (0.9)	40.2	
>65 years	277	7.9 (0.6)	264	8.1 (0.5)	2.5	
**Gender**						0.81
Male	1263	53.8 (1.0)	1276	53.4 (1.1)	-0.7	
Female	1538	46.2 (1.0)	1447	46.6 (1.1)	0.8	
**Race/Ethnicity**						0.25
Non-Hispanic white	2339	75.2 (1.1)	2162	73.5 (1.1)	-2.2	
Hispanic/Latino	138	9.0 (1.0)	175	10.6 (0.8)	17.7	
African American	236	12.5 (0.8)	257	11.4 (0.7)	-9.0	
Asian/PI	47	2.3 (0.5)	76	3.1 (0.4)	33.8	
Other	41	1.1 (0.3)	53	1.5 (0.2)	40.2	
**Education**						0.31
Less than high school	492	20.2 (1.2)	437	18.3 (1.0)	-9.5	
High school	1143	38.7 (1.2)	1096	39.3 (1.1)	1.4	
Some college	777	27.8 (1.2)	817	30.2 (1.1)	8.6	
College graduate	389	13.3 (0.9)	373	12.3 (0.8)	-7.8	
**Income**						**<0.0008**
Below federal poverty line	426	17.1 (1.3)	496	20.4 (1.1)	19.0	
100%-<200% federal poverty line	554	20.9 (1.1)	680	25.5 (1.0)	21.8	
200-<300% federal poverty line	514	19.5 (1.1)	476	18.0 (1.0)	-7.4	
≥300% federal poverty line	1125	42.5 (1.5)	1070	36.1 (1.1)	-15.0	
**Consumption at baseline**						**<0.0005**
0–9 cigarettes per day	720	29.4 (1.3)	824	33.2(1.2)	13.1	
10–19 cigarettes per day	710	27.0 (1.4)	824	30.3 (1.1)	12.5	
≥ 20 cigarettes per day	1320	43.7 (1.0)	1079	36.1 (1.1)	-16.5	
**Smoke-free Home**						**< .0001**
No smoke-free home	2086	72.0 (1.2)	1527	54.7 (1.2)	-24.1	
Smoke-free home	715	28.0 (1.2)	1196	45.3 (1.2)	62.0	
**Tobacco Tax** ^a^						**< .0001**
Lowest tertile	773	29.7 (1.1)	747	27.3 (1.3)	-8.3	
Middle tertile	1042	30.4 (1.1)	1194	49.6 (1.4)	63.2	
Highest tertile	986	39.9 (1.2)	782	23.1 (1.0)	-42.0	
**State Tobacco Control Expenditure** ^b^						**< .0001**
Lowest quartile	642	22.7 (1.1)	766	34.0 (1.3)	49.8	
2^nd^ quartile	734	21.3 (1.0)	661	24.6 (1.1)	15.4	
3^rd^ quartile	707	27.9 (1.2)	724	32.6 (1.3)	17.0	
4^th^ quartile	718	28.1 (1.3)	572	8.8 (0.5)	-68.6	

^**a**^ In 2002, lowest tertile had an adjusted tax $0.03 to $0.28, the middle tertile had an adjusted tax $0.30 to $0.65, and the highest tertile had an adjusted tax $0.70 to $1.87. In 2010, the lowest tertile had an adjusted tax $0.07 to $0.81 cents, the middle tertile had an adjusted tax $0.82 to $1.65, and the highest tertile had an adjusted tax $1.70 to $3.56.

^**b**^In 2002, the lowest quartile spent <11% of recommended, the 2nd quartile spent <39%, and the 4th quartile spent >63.1%. In 2010, the lowest quartile spent <5.1%, the 2nd quartile spent <15% and the 4th quartile spent >33%.

^c^ P-value testing the difference between the 2 surveys

SE: Standard error; CI: Confidence interval

### Differences in smoke-free homes by income level between the two longitudinal surveys

**Fig 1:**
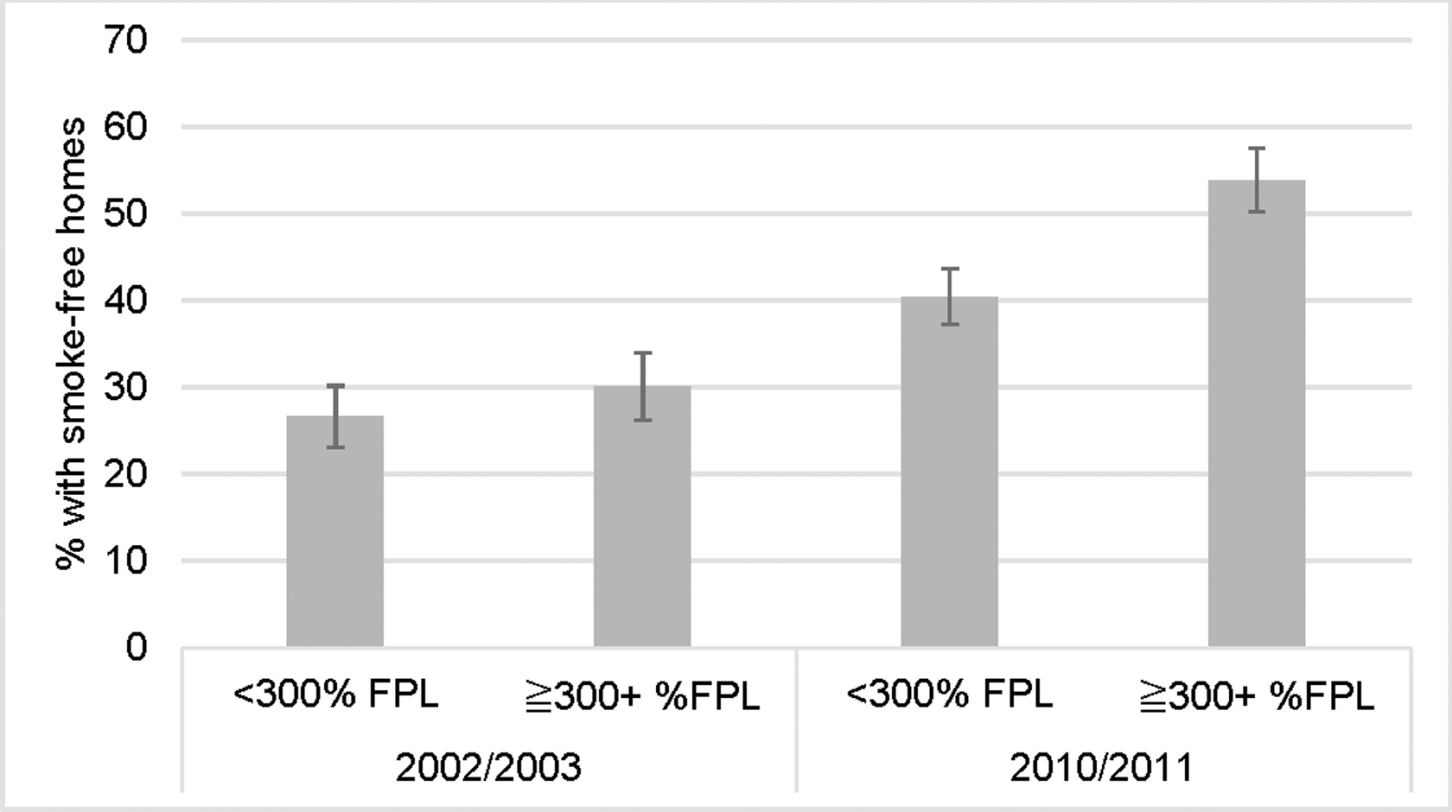
Smoke-free homes by income level in 2002/2003 and 2010/2011 (TUS-CPS).

In the nine years between these two longitudinal studies, the prevalence of smoke-free homes increased, however there were differences by income level. In 2002, there was no difference in the prevalence of smoke-free homes between smokers living <300% of the FPL (26.6%, 95% CI 23.1–30.1) and those living ≥ 300% FPL (30.1%, 95% CI 26.2–33.9, p = 0.2). However, in 2010, the prevalence of smoke-free homes was 33.1% lower among smokers living <300% of the FPL (40.4%, 95% CI 37.2–43.7) compared to those living at ≥ 300% FPL (53.8%, 95% CI 50.2–57.5, p<0.0001)**(Fig 1)**.

### Association of smoke-free homes and 1+day quit attempts

The probability that a smoker would report a quit attempt between the baseline and follow-up surveys did not vary by survey year or the following characteristics of smokers: gender, race-ethnicity, and educational level (**Table 2)**. Smokers who smoked 10–19 cigarettes per day (40.2% vs. 43.6%, Adjusted Odds Ratio [AOR] 0.8, 95% CI 0.6–0.9) or who smoked ≥ 20 cigarettes per day (32.7% vs. 43.6%, AOR 0.6, 95% CI 0.5–0.7) were less likely to make a quit attempt. Smokers with a smoke-free home were more likely to report a quit attempt than those without (39.2% vs. 32.7%, AOR 1.3, 95% CI 1.0–1.5). There was no interaction between smoke-free homes and cigarette consumption (p = 0.9) and smoke-free homes and income (p = 0.4).

**Table 2:** Association of smoke-free homes and 1+-day quit attempts ^a^^,^^b^ (TUS-CPS).

	Quit Attempts During year		
	% (SE)	Adjusted Odds Ratio (95% CI)	P-Value
**Survey Year**			
2002/2003	38.2 (1.2)	Ref.	
2010/2011	38.3 (1.1)	1.0 (0.8–1.1)	0.22
**Age**			
18–29 years	41.7 (2.3)	Ref.	
30–49 years	38.9 (1.0)	0.9 (0.7–1.1)	0.15
50–64 years	36.7 (1.4)	0.8 (0.6–1.0)	0.04
≥65 years	29.1 (2.3)	0.5 (0.4–0.7)	**<0.001**
**Sex**			
Female	42.0 (1.1)	Ref.	
Male	34.9 (1.2)	0.9 (0.7–1.0)	0.05
**Race/Ethnicity**			
Non-Hispanic white	38.7 (0.9)	Ref.	
Hispanic/Latino	33.8 (3.0)	0.7 (0.5–0.9)	0.08
African American	39.7 (2.9)	1.0 (0.8–1.4)	0.15
Asian/PI	35.0 (5.7)	0.8 (0.5–1.4)	0.81
Other	35.6 (5.9)	0.8 (0.5–1.5)	0.91
**Education**			
Less than high school	33.5 (2.0)	Ref.	
High school	38.1 (1.3)	1.1 (0.9–1.4)	0.51
Some college	41.0 (1.5)	1.2 (0.9–1.5)	0.26
College graduate	39.4 (2.3)	1.1 (0.8–1.4)	0.78
**Income**			
< 300% federal poverty line	39.1 (1.5)	Ref.	
≥300% federal poverty line	37.7 (1.3)	0.8 (0.7–0.9)	**<0.02**
**Consumption**			
0–9 cigarettes per day	43.6 (1.6)	Ref.	
10–19 cigarettes per day	40.2 (1.8)	0.8 (0.6–0.9)	**<0.0092**
≥ 20 cigarettes per day	32.7 (1.3)	0.6 (0.5–0.7)	**<0.0001**
**Smoke-free Home**			
No smoke-free home	32.7 (1.3)	Ref.	
Smoke-free home	39.2 (1.5)	1.3 (1.0–1.5)	**<0.01**

^a^ Controlled for state tertile of cigarette tax, state estimate of expenditures on tobacco control, and doctor’s advice to quit.

^b^ p-value for interaction between smoke-free homes and cigarette consumption was p = 0.9 and smoke-free homes and income was p = 0.4.

SE: Standard error; CI: Confidence interval

### Association of smoke-free homes and 30+days abstinence

Smokers in the 2010 cohort were more likely to be 30+days abstinent at follow-up than smokers in the 2002 cohort (20.6% vs. 15.5%, AOR = 1.5, 95% CI 1.1–2.0), after adjusting for age, gender, race/ethnicity, education and other predictors of cessation (**Table 3**). Smokers with incomes ≥300% of poverty level were more likely to be 30+-days abstinent than those who were below the poverty line (24.4% vs.13.8, AOR = 1.9, 95% CI 1.4–2.7). Smokers who smoked ≥ 20 cigarettes per day were less likely to achieve 30+days abstinence compared to those who smoked 0–9 cigarettes per day (12.3% vs. 23.2%, AOR 0.7, 95% CI 0.5–0.9). Smokers who had a smoke-free home (24.6% vs. 13.6%, AOR 1.6, 95% CI 1.2–2.1) were more likely to be 30+days abstinent than those without a smoke-free home. There was no interaction between smoke-free home and cigarette consumption (p = 0.3) and smoke-free homes and income (p = 0.3).

**Table 3:** Association of smoke-free home and 30+days abstinence ^a^^,^^b^ (TUS-CPS).

	Quit Attempts During year		
	% (SE)	Adjusted Odds Ratio (95% CI)	P-Value
**Survey Year**			
2002/2003	15.5 (1.4)	Ref.	
2010/2011	20.6 (1.5)	1.5 (1.1–2.0)	**<0.008**
**Age**			
18–29 years	20.2 (2.7)	Ref.	
30–49 years	17.1 (1.4)	0.8 (0.5–1.2)	0.31
50–64 years	16.1 (1.6)	0.9 (0.5–1.3)	0.48
≥65 years	21.6 (4.0)	1.5 (0.8–2.8)	0.25
**Sex**			
Female	18.1 (1.3)	Ref.	
Male	17.7 (1.7)	0.9 (0.6–1.2)	0.40
**Race/ethnicity**			
Non-Hispanic white	18.0 (1.1)	Ref.	
Hispanic/Latino	22.7 (4.3)	1.2 (0.7–2.1)	0.25
African American	12.2 (2.7)	0.8 (0.5–1.3)	0.74
Asian/PI	32.9 (12.1)	1.7 (0.7–4.3)	0.12
Other	5.5 (4.4)	0.3 (0.05–1.9)	0.16
**Education**			
Less than high school	14.2 (2.4)	Ref.	
High school	14.5 (1.4)	0.9 (0.6–1.4)	0.07
Some college	21.3 (1.9)	1.3 (0.8–2.2)	0.16
College graduate	24.6 (3.5)	1.2 (0.7–2.1)	0.46
**Income level**			
< 300% federal poverty line	13.7 (1.3)	Ref.	
≥300% federal poverty line	24.4 (1.8)	1.9 (1.4–2.7)	**<0.0001**
**Consumption**			
0–9 cigarettes per day	23.2 (2.1)	Ref.	
10–19 cigarettes per day	17.3 (1.9)	0.8 (0.6–1.2)	0.4
≥ 20 cigarettes per day	12.3 (1.3)	0.7 (0.5–0.9)	**<0.03**
**Smoke-free homes**			
No smoke-free home	13.6 (1.1)	Ref.	
Smoke-free home	24.6 (2.8)	1.6 (1.2–2.1)	**< .0021**

^a^Controlled for state tertile of cigarette tax, state estimate of expenditure on tobacco control, doctor’s advice to quit, and use of pharmaceutical aid during the last quit attempt.

^b^ p-value for interaction between smoke-free homes and cigarette consumption was p = 0.3 and smoke-free homes and income was p = 0.3

SE: Standard error; CI: Confidence interval

### Mediation analysis to examine whether smoke-free homes contribute to income disparities in 30+days abstinence

Individuals living ≥ 300% FPL were significantly more likely to achieve 30+days abstinence compared to those living < 300% FPL (AOR 2.0, 95% C.I. 1.5–2.7), after adjusting for confounders and after not including smoke-free homes in the model. The CDE and NIE were 1.9 (95%CI 1.72–2.14) and 1.3 (95%CI 1.18–1.36) respectively (Table 4). This corresponds to a proportion mediated of 36% (95% CI 28.1–43.1), which means that the increase in smoke-free homes between the surveys explained 36% of the income disparity in 30+days abstinence. Said differently, our findings suggest that by intervening to set a similar change in smoke free homes between both income groups we could reduce the income disparity in 30+days abstinence by 36%.

**Table 4:** Mediation analysis^a^ with smoke-free homes as a contributor to income disparity in 30+days abstinence (TUS-CPS).

AOR (95% CI) for Total effect	AOR (95% CI) for Controlled direct effect	AOR (95% CI) for Natural indirect effect	Proportion mediated % (95% CI)
2.47 (2.20–2.81)	1.94 (1.72–2.14)	1.27 (1.18–1.36)	36.0 (28.1–43.1)

^a^ Controlled for age, sex, race/ethnicity, education, income level, consumption, state tertile of cigarette tax, state estimate of expenditure on tobacco control, doctor’s advice to quit, and use of pharmaceutical aids during the last quit attempt.

CI: Confidence interval

## Discussion

In the nine years between these two longitudinal surveys, the smoking population got older and poorer, with a 40% increase in smokers who were ≥50 years of age and living below 200% of the FPL. Smokers were less likely to live in states that were leaders in tobacco control, with a 42% decrease in smokers living in states with the highest taxes and a 68% decrease in states with the highest tobacco control expenditures. At the same time, smoking intensity declined and the proportion of the smoking population who were medium-to-light smokers (< 1 pack/day) and who had a smoke-free home increased to over a third of the smoking population. However, there was a marked disparity in the adoption of smoke-free homes among those with lower incomes. In 2010, smokers with lower incomes were 33% less likely to report a smoke-free home compared to those with higher incomes, suggesting that the income disparity in the adoption of smoke-free homes increased over time. However, the difference in smoke-free homes between the surveys explained up to 36% of the income disparity in 30+days abstinence between lower and higher income smokers. These findings underscore the importance of smoke-free homes as an effective cessation mediator among low-income smokers, with a potential to reduce income disparities in smoking behaviors.

The three primary predictors of 30+days abstinence were having a higher income, a lower consumption, and having a smoke-free home; however there was no significant interaction between smoke-free homes and income or smoke-free homes and cigarette consumption. These findings were observed after adjusting for other known predictors of cessation such as smoking behavior, tobacco control policies, and receipt of cessation services. The absence of an interaction suggests that, when adopted, a smoke-free home could increase successful cessation, irrespective of income level or intensity of cigarette consumption.

The finding that 30+days abstinence increased between 2003 and 2011 in the absence of an increase in quit attempts, another known predictor of cessation, contradicts suggestions from a previous study that the major way to increase successful cessation was to increase quit attempts.[35] The greater probability of 30+days abstinence among those with higher incomes is unlikely to explain the population increase in this variable between 2003 and 2010 as, over that same time period, smoking became more characteristic of lower income populations.[4] Instead, there is good evidence to postulate that the increase in smoke-free homes and the reduction in cigarette consumption that occurred among all income groups could have contributed to this effect.[8, 22, 36] Smokers who voluntarily implement a smoke-free home are more likely to be lighter smokers prior to this decision. [7] Further, as this home rule makes it more difficult to smoke *ad libitum*, it is possible that the implementation of a smoke-free home leads to a reduction in smoking intensity prior to a quit attempt.[11] Smoke-free homes also pose challenges to smoking previously favorite cigarettes (e.g. after a meal, first cigarette in the morning), potentially reducing relapse back to smoking.[8, 36]

The slower diffusion of smoke-free homes among low-income smokers could be related to pervasiveness of pro-tobacco social norms in poor neighborhoods[15], including a higher prevalence of tobacco advertising.[37] Lack of agency to negotiate the adoption of a smoke-free home is another common barrier among low-income smokers.[38] Our findings lend particular salience to HUD’s rule to implement smoke-free policies in public housing.[13] This rule, enacted in January 2016, mandated public housing authorities to implement indoor smoke-free policies in all of their buildings by July 2018.[13] This policy, which is expected to impact over 1.2 million low-income households and over 700,000 children, the majority from racial/ethnic minority groups who are disproportionately exposed to secondhand smoke, presents an unprecedented opportunity to reduce disparities related to tobacco use.[13] Our findings that smoke-free homes could potentially reduce a third of the income disparity in cessation outcomes highlights the potential benefits of HUD’s smoke-free policies in reducing tobacco-related harm by increasing cessation among low-income populations. Future studies could take advantage of this natural experiment to examine the longitudinal effects of this policy on reducing the gap in cessation as well as tobacco-related morbidity and mortality among low-income populations residing in public housing in the U.S.

A strength of this study is that it uses data from a large nationally representative survey that allows population estimates of behavior. Respondents did not expect to complete a second Tobacco Use Supplement at the follow-up survey, thus limiting attrition related to the topic of the survey. The study also includes causal mediation analysis to quantify the effects of smoke-free homes on cessation outcomes between lower and higher income smokers.

### Limitations

However, there are also several limitations. An observational study limits our ability to make causal inferences. All data are self-reported which could lead to misclassification bias; however the likelihood of this bias is low given that prior studies have validated self-reported smoking status in population surveys.[39] After a quit attempt, it is well known that most relapse within the first month and by limiting our main outcome to persistent abstinence of at least 30 days, we have accounted for that. Using such a measure, in preference to a longer abstinence criterion, has the advantage of including more recent quit attempts in a 12 month follow-up study. By pooling data, we were able to increase power in our analysis but were unable to assess between-subjects trends in quit attempts and 30+days abstinence by income level during the study time period.

In summary, increasing the diffusion of smoke-free homes among low-income smokers could potentially mitigate the income disparity in successful cessation. Implementing smoke-free policies in low-income, multi-unit housing is one strategy to increase the diffusion of smoke-free homes among low-income populations in the U.S.
