# Correction: Income disparities in smoking cessation and the diffusion of smoke-free homes among U.S. smokers: Results from two longitudinal surveys

**DOI:** 10.1371/journal.pone.0208153

**Published:** 2018-11-21

**Authors:** Maya Vijayaraghavan, Tarik Benmarhnia, John P. Pierce, Martha M. White, Jennie Kempster, Yuyan Shi, Dennis R. Trinidad, Karen Messer

The second author’s name is spelled incorrectly. The correct name is: Tarik Benmarhnia. The correct citation is: Vijayaraghavan M, Benmarhnia T, Pierce JP, White MM, Kempster J, Shi Y, et al. (2018) Income disparities in smoking cessation and the diffusion of smoke-free homes among U.S. smokers: Results from two longitudinal surveys. PLoS ONE 13(7): e0201467. https://doi.org/10.1371/journal.pone.0201467
